# Characterization of Protein Complexes and Subcomplexes in Protein-Protein Interaction Databases

**DOI:** 10.1155/2015/245075

**Published:** 2015-02-04

**Authors:** Nazar Zaki, Elfadil A. Mohamed, Antonio Mora

**Affiliations:** ^1^Intelligent Systems, College of Information Technology, UAEU, Al Ain 17551, UAE; ^2^Department of Management Information Systems, Al Ain University of Science and Technology, Al Ain, UAE; ^3^Laboratory of Integrative Systems Medicine (LISM), CNR, Pisa, Italy

## Abstract

The identification and characterization of protein complexes implicated in protein-protein interaction data are crucial to the understanding of the molecular events under normal and abnormal physiological conditions. This paper provides a novel characterization of subcomplexes in protein interaction databases, stressing definition and representation issues, quantification, biological validation, network metrics, motifs, modularity, and gene ontology (GO) terms. The paper introduces the concept of “nested group” as a way to represent subcomplexes and estimates that around 15% of those nested group with the higher Jaccard index may be a result of data artifacts in protein interaction databases, while a number of them can be found in biologically important modular structures or dynamic structures. We also found that network centralities, enrichment in essential proteins, GO terms related to regulation, imperfect 5-clique motifs, and higher GO homogeneity can be used to identify proteins in nested complexes.

## 1. Introduction

Protein complexes are in the center of various biological functions in the cell and, therefore, several disorders are believed to be consequences of changes in a single protein subunit, and, thus, in its set of associated partners and functionality. The identification and characterization of protein complexes and subcomplexes are crucial to the understanding of the molecular events under normal and disease conditions [1]. For instance, Vanunu et al. [2] used data on 1,369 diseases such as prostate cancer, Alzheimer's, and type 2 diabetes mellitus from the OMIM knowledge-base, and they were able to rank the true causal gene for 34% of the diseases and infer 139 disease-related complexes that are highly coherent in terms of the function, expression, and conservation of their member proteins.

A subcomplex can be defined as a functional complex which is a subset of a larger functional complex. Intuitively, we could think of two sets of proteins where the protein subunits of the subcomplex are a subset of the protein subunits of a larger complex. However, a subcomplex can also be defined as cluster lying inside a larger network cluster, that is, the most dense region inside a larger connected regions. As an example, Sales-Pardo et al. [3] presented a method to extract hierarchical organization, which identifies different levels of network organization. This method does not work with overlapping information and if their hierarchies can predict the real nesting structure found in interaction databases is yet to be proven. Other authors paid more attention to the “cores” that repeat in several complexes and the “attachments” that make them different to each other [4]. In this case, the core of a core-attachment structure could be considered as a subcomplex. A similar approach focuses on studying multiclustered and monoclustered proteins after applying overlapping clustering algorithms to protein interaction networks; subcomplexes can be understood as multicluster structures [5]. Together with all these approaches, the subcomplex term is also used to strictly define a biologically relevant subcomplex; that is, the subcomplexes that have been experimentally found to be functional and independent from the complex that contains them.

A few studies have discussed the properties of subcomplexes in protein interaction networks. Becker et al. [5] discussed the properties of proteins in overlapping complexes showed that degree and centralities, as well as the involvement of a protein in cancer, are useful to distinguish between monoclustered (nonoverlapping) and multiclustered (overlapping) proteins generated by the OCG algorithm. Multiclustered proteins have higher degree and centralities than monoclustered, and “products of cancer genes are also enriched among multi-clustered proteins” [5]. At the same time, multiclustered proteins are, on average, annotated to more terms than monoclustered and are enriched in biological process GO terms referring to regulatory functions and protein complex assembly [6, 7]. Hollunder et al. [8] presented an early discussion of properties of subcomplexes, defined as statistically significant groups of proteins repeated inside in bigger complexes. The authors claimed that subcomplexes are functionally and spatially more homogeneous than the complexes that contain them and that they are enriched in essential proteins. Finally, the “linkcomm” R package [9] includes a detector of “nested communities,” which could be understood as a community inside another community, where both have good modularity values. However, it is yet to be proven that “nested communities” are related to biological subcomplexes.

There are at least two problems with the previous approaches. The first one is that they do not take into account higher order nesting (sub-sub-complexes) and overlap between multiple subcomplexes. The second one is that the conclusions are extracted from specific protein complex data sets but not from a consolidated database of complexes. Integrated protein interaction databases offer the most complete source of complexes and subcomplexes, but this comes to the price of introducing several different sources of noise. Subcomplexes in databases may have a biological role as, for example, building blocks of a bigger structure, but can also be due to experimental or curation errors.

Protein complex data usually comes from a group of copurified proteins which, in order to be recorded in an interaction format such as the PSI-MITAB format [10] or to be included in a protein interaction network, are usually represented as either matrix models (where all possible interactions between their nodes are assumed to exist) or, more commonly, spoke models (where one protein is chosen to be a central hub that links to all other proteins) [11, 12]. In order to choose the hub protein of the spoke model, it is a common practice to choose the bait used in the original experiment; however, when the experimental method uses no bait or there is no bait information in the source database, analysts can choose a random protein of the copurified group as the hub. The effect of this decision is that, when maximal complex and subcomplex are represented as matrix models or as spoke models with the same bait, all nodes and edges of the subcomplex will be subsets of those of the maximal complex. In these cases, it is common that prediction algorithms and visualization software detect only the maximal complex. However, if maximal complex and subcomplex are represented as spoke models with a different bait, or if the bait of the subcomplex does not detect the hub of the maximal complex, the subset of edges of the subcomplex will be different from the edges of the maximal complex, and protein complex and subcomplex will not look as a hierarchical organization but as the overlap of two complexes with different hubs.

For the abovementioned reasons, we have created the concept of a “nested group” of complexes. We define a nested group as the group formed by one complex and all its subcomplexes. A subcomplex was defined as a complex whose nodes are a subset of the nodes of a larger complex (which we call it “maximal complex”), while its edges do not need to be a subset of the maximal complex edges. Nested groups of complexes take into account the hierarchical and overlapping nature of complexes, that is, the existence of sub-sub-complexes and overlapping subcomplexes inside a maximal complex. At the same time, complexes do not appear hierarchical or overlapping or both in protein interaction databases depending on representation issues, as a nested group is defined in terms of nodes and not edges.

In [12], the authors thoroughly discussed the problem of generating complexes and subcomplexes, and they reviewed different protein complex/subcomplex prediction methods. In this paper, we are interested in the properties of those generated subcomplexes when studied as a whole in protein interaction databases. Here we start a systematic study of nested complexes and their properties, in order to validate the problem and identify strategies that could be useful in a nested complex prediction algorithm. First we start defining and quantifying complex nesting in human interactome, then we explore the best ways to represent it and visualize it, and then we analyze a series of properties of the networks, which may help to distinguish nested from nonnested complexes. We compare our findings regarding these properties with previous reports of properties of overlapping proteins [5] and earlier definitions of nested complexes [8, 13].

## 2. Methods

All analysis in this paper was performed using R v.2.15.2 and some of its packages, including “iRefR” v.1.00 [11], “igraph” v.0.64 (http://cran.r-project.org/web/packages/igraph/index.html), “org.Hs.eg.db” v.2.8.0 (http://www.bioconductor.org/packages/release/data/annotation/html/org.Hs.eg.db.html), and “NeMo” v.1.0.1 (http://artax.karlin.mff.cuni.cz/r-help/library/NeMo/html/00Index.html). Source codes and data sets are available from http://faculty.uaeu.ac.ae/nzaki/Research.htm.

### 2.1. Data

#### 2.1.1. Interaction Data

The package iRefIndex [14] was chosen as the data source for interaction data. iRefIndex is a consolidated database of 13 of the main protein interaction databases. The iRefIndex keeps provenance information, which allows the user to select only the records that belong to a given source database or paper. Moreover, the most important feature of iRefIndex compared to other resources is that iRefIndex is nonredundant. iRefIndex reported that, before consolidating the 13 databases, 46% of the current available information was in fact redundant. This way, iRefIndex is the largest nonredundant repository of protein interactions. iRefIndex also includes information of confidence scores and experimental methods. The human iRefIndex database v.9.0 contains around 401,140 records (PSI-MITAB format), which correspond to 347,753 records of human-human interactions, and 289,451 records excluding predicted interactions. iRefIndex can be manipulated using an R package “iRefR” [11] or a Cytoscape plugin “iRefScape” [15]. “iRefR” is a pipeline between iRefIndex and some network analysis packages (igraph, graph, RBGL, and others). Using iRefR, we extracted a network containing all iRefIndex records that have been reported to the primary databases as protein complexes (iRef_C). In the 289,451 abovementioned records, we found 58,854 complex records, which correspond to 5,701 unique human complexes. A network of these complexes, using a spoke representation, contains 7,468 nodes and 37,075 edges. Most proteins belong to a giant connected component containing 98,3% of all proteins, while the rest are divided into 35 small connected components. Most complexes have 3 or 4 proteins, while a few complexes can have sizes up to 220 proteins. The average size of protein complexes in this network is 7.24 proteins.

#### 2.1.2. Cancer Data

OMIMs Morbid Map [16] is one of the most popular resources to link disease information to gene information. The subset of OMIMs cancer information has been constructed by selecting the records containing at least one of 20 cancer-related keywords such as cancer, carcinoma, melanoma, and leukemia. There are a total of 455 cancer-related proteins out of a total of 1,941 disease-related genes.

#### 2.1.3. CORUM and MIPS Data

CORUM complexes and curated yeast complexes were downloaded from the MIPS website [17].

### 2.2. Listing and Visualizing All Nested Groups

This task makes use of the subset of known complexes previously extracted from iRefIndex (iRef_C). A “nested group” of complexes was defined as a group composed of a maximal complex and subcomplexes formed by some of the subunits of the larger one. In order to identify these groups, it is important to take into account that some of the nested complexes can overlap and, moreover, some of the nested complexes can have nested complexes at the same time. Therefore, we started the identification process from a table with all pairs of complexes with a meet-min index [12, 18] of 1 (i.e., one being a subset of the other) and ordered them by placing the complex with a higher number of nodes in the first column followed by constructing a directed graph for such an edge list.

The overlap matrix was constructed using the length of the intersection between the subunits of every pair of complexes. The nesting matrix was constructed using the length of the meet-min index. The meet-min index is calculated as follows:

(1) $$\begin{array}{c} \text{Meet-min}=\frac{N\left(C1\cap C2\right)}{\min\left(N\left(C1\right),N\left(C2\right)\right)}, \end{array}$$

where *C*1 and *C*2 represent Complex-1 and Complex-2, which are lists of protein subunits, *N* represents the number of elements in each list, and min⁡ indicates the smallest of the two values. The Jaccard index is computed as follows:

(2) $$\begin{array}{c} \text{Jaccard}=\frac{N\left(C1\cap C2\right)}{N\left(C1\cup C2\right)}. \end{array}$$

**Figure 1:**
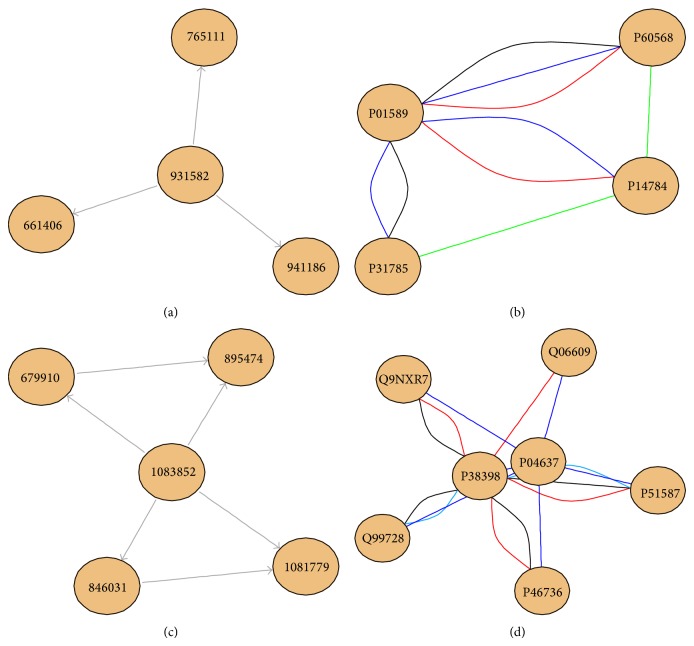
Nested protein complexes from iRefIndex. (a) Group ID: 421, complex plot. Labels correspond to iRefIndex ROG IDs. (b) Group ID: 421, protein plot. Labels correspond to UniProt IDs; colors denote different complexes and multiple edges representing membership to multiple complexes. P01589: interleukin-2 receptor subunit alpha, P60568: interleukin-2, P31785: cytokine receptor common subunit gamma, and P14784: interleukin-2 receptor subunit beta. (c) Group ID: 570, complex plot. (d) Group ID: 570, protein plot. Q9NXR7: BRCA1-A complex subunit BRE, Q06609: DNA repair protein RAD51 homolog 1, P38398: breast cancer type 1 susceptibility protein, P04637: cellular tumor antigen p53, Q99728: BRCA1-associated RING domain protein 1, P46736: Lys-63-specific deubiquitinase BRCC36, and P51587: breast cancer type 2 susceptibility protein. Arrows indicate a subsumption relationship.

Depth first search algorithm was used on the resulting graph to find groups of all nodes that can be reached from the nodes of origin and evaluate the corresponding meet-min score again to remove redundant groups. As a result of this, we found 1427 nested groups (groups of a maximal complex and one or more subcomplexes). We have also written R codes to plot and visualize the previous nested groups, based on the “iRefR” and “igraph” packages. These functions can plot two kinds of plots per nested group: the first plot is a directed graph showing which complexes are a subset of which others, while the second plot is showing all protein subunits in a subgraph of the interaction network.  Figure 1 shows both plots for two nested groups from iRefIndex. The group 570 is formed by five complexes (Figure 1(c)), where four of them are subsets of one central hub, in the shape of a double-cycle motif. The group 421 is a group of four complexes (Figure 1(a)), where one contains the other three, in the form of a hub motif (central node connected to multiple nodes with low degree).  Figure 1(b) shows that these four complexes contain four proteins, and an additional detail appears here: while two of these complexes (the ones with red and black edges) share the same hub/bait with the maximal complex (blue edges complex), the complex with green edges was chosen for a different hub protein in an experiment that does not detect the hub of the maximal complex; therefore, in a graph with single edges, the black and red complexes (hierarchical) would not be seen, while the green one would look as a different overlapping complex. This way, most algorithms and visualization software might work with these artificial motifs found in databases due to representation issues, while in fact the only certain knowledge we have about those complexes is that they share or do not share some subunits (nodes), without valid information regarding their edges.

The overlap matrix was constructed using the length of the intersection between the subunits of every pair of complexes. The nesting matrix was constructed using the length of the meet-min index [18].

### 2.3. Network Analysis and Relationship to Cancer in Nested Groups

The degree is the number of edges linking to a node or, in this case, the number of interactions for a given protein. The betweenness centrality is a measure of the number of shortest paths that cross a given node or protein. The page rank centrality uses an implementation of Google's PageRank algorithm [19, 20]. For these computations, the “igraph” R package was used. The “org.Hs.eg.db” R package was used to find the GO terms related to the given gene IDs. A routine to count the number of GO terms, as well as to compute GO term homogeneity between the proteins of a complex (using the Jaccard index), was written.

The global and local clustering coefficients were calculated. The global clustering coefficient is defined as the number of closed triplets over the total number of triplets. An open triplet is three nodes connected by two edges, while a closed triplet is three nodes connected by three edges. The local clustering coefficient which was calculated using “igraph” is the proportion of edges between the nodes in the neighborhood of a query node, divided by the number of possible edges between them.

The Wilcoxon and hypergeometric tests were also calculated. The Wilcoxon test is a statistical-hypothesis test which can be used to compare two samples and evaluate if their population means differ. The null hypothesis is that the median difference between data from both samples is zero. A *P* value smaller than the significance level (here, 0.05) means that the null hypothesis should be rejected, that is, the difference between samples is statistically significant. The hypergeometric test takes into account the size of the population, the size of the successful population, the size of the sample, and the size of the successful sample, translated into a *P* value that describes the probability of this number of successes given such a background. *P* values smaller than a threshold (here, 0.05) are accepted as evidence of overrepresentation. In order to examine all possible motifs involving 3, 4, and 5 nodes together, the R “NeMo” package was used.

### 2.4. Modularity Analysis in Nested Groups

Modularity is a measure of how good a clustering process is. Newman-Girvan's modularity was computed using the “igraph” R package. The community detection algorithms used include edge betweenness communities, walktrap communities, and label propagation communities. Community detection computations were performed using the “igraph” R package. Precision is defined as the ratio of true positives to all positives (true and false positives), while FDR is defined as the ratio of false positives to all positives.

### 2.5. Gene Ontology Analysis in Nested Groups

In order to count GO terms in nested groups, we used “org.Hs.eg.,” “GO.db,” and “annotate” R packages.

### 2.6. Essentiality Analysis in Nested Groups

A list of essential genes was extracted from the DEG database [21] and translated to proteins using DAVID [22].

## 3. Results and Discussion

### 3.1. Descriptive Analysis

#### 3.1.1. Bibliometric Validation of Biologically Relevant Nested Complexes

It is possible to hypothesize that the main reason of complexes and subcomplexes exist in interaction databases is merely that some studies found an incomplete version of the complex while other studies found the complete one. It is possible that two databases report the same complex from the same paper but one of them made a mistake, leaving one or more subunits out of the complex. In such cases, the nesting problem would be an artifact and the smaller (incomplete) complexes should be discarded. On the other hand, it is very well known that some biological processes and structures are composed of subcomplexes integrating into bigger complexes. Therefore, a first necessary step is to find ways to quantify these two cases and prove if the nesting problem is rather relevant. In order to do that, we performed a bibliometric validation, exploring the iRefIndex database [14] in order to identify nested complexes found in the same paper and the same database. This would be an indication that the authors of the paper were aware of the existence of both the complex and the subcomplex (nested complex), and the difference between their subunits is not a result from a different study or from a mistake in a database. An evaluation of the set of 1,427 nested groups of the human interactome showed that all the complexes in 189 out of the 1,427 generated nested groups were entirely coming from the same paper and the same database, which can be taken as a first rough indication of a minimum amount of nested groups with biological relevance (approximately 13% of the cases).

A second approach is to find annotation information of all the complexes in a nested group and decide whether biological relevance existed. In order to do this, we evaluated two different curated databases of complexes: CORUM (mammalian) and MIPS (yeast). The CORUM database [23] is a manually curated database of complexes which contains functional annotation. CORUM includes information regarding specific biological functions, structures, or diseases, which could be used as a validation of the relevance of the nesting problem. Following the same algorithm that we previously used, 750 nested groups were generated from this database. In CORUM, we reviewed three specific biological examples: the nuclear pore complex, which is a structure known to be formed by several subcomplexes, and the complexes involved in cancer and the DNA-repair process. A simple text-search of the term “pore” identified that the term is present in one nested group (Group ID: NID_43), which is formed by three complexes. This shows that there are multiple cases in which nesting exists, such as a substructure of a macrostructure (nuclear pore complex); in functional processes such as DNA repair (27 different examples); or in diseases such as cancer [24] (32 different examples). This suggests that nesting might not only be a database problem, or a source of noise for community detection algorithms, but a relevant biological phenomenon.

A more comprehensive validation study was done using the MIPS set of yeast complexes, which is a curated data set that has been used as a gold standard in high-throughput complex detection studies. The set contains 16 nested pairs, grouped in 11 nested groups. Some interesting examples with meet-min equal to 1 include the following.

- The TIM22 complex (TIM10, TIM12, TIM22, TIM54, TIM9) is involved in the integration of carrier preproteins into the inner membrane, which appears as a maximal complex, with the TIM9-TIM10 complex (TIM10, TIM9) as a subcomplex. TIM9-TIM10 is a hexamer that directs preproteins to the inner mitochondrial membrane in order to interact with the TIM22 complex. Around 95% of TIM9-TIM10 has been reported to be in the intermembrane space and may help stabilizing carrier preproteins, while 5% is linked, via TIM12, to the outer surface of the TIM22 complex.
- On the other hand, the Transcription Factor II H (TFIIH), involved in transcription initiation and nucleotide excision repair, consists of a core complex (RAD3, SSL1, SSL2, TFB1, TFB2, TFB4) connected to the cyclin-activating kinase subcomplex (CCL1, KIN28, TFB3), but, at the same time, is a subcomplex of the Transcription preinitiation complex, which contains RNA polymerase II, TFIIA, TFIIB, TFIID, TFIIE, TFIIF, and TFIIH, plus additional regulatory complexes.
- The regulatory histone acetylation complexes (HATs) are good examples of both nesting and overlapping: the SAGA complex (ADA2, ADA3, CHD1, GCN5, HFI1, SGF11, SGF29, SGF73, SPT20, SPT3, SPT7, SPT8, SUS1, TAF10, TAF12, TAF5, TAF6, TAF9, TRA1, UBP8) acetylates histone H3, among other roles. The SALSA complex (ADA2, ADA3, GCN5, HFI1, SPT20, SPT3, SPT7, TAF12, TAF5, TAF6, TRA1) is one functional subcomplex, annotated in UniProt as an altered form of SAGA. The SLIK complex (ADA2, ADA3, CHD1, GCN5, HFI1, RTG2, SGF29, SPT20, SPT3, SPT7, TAF10, TAF12, TAF5, TAF6, TAF9, TRA1, UBP8) highly overlaps with SAGA and their functions are partially redundant. Finally, the ADA/GCN5 complex (ADA2, ADA3, GCN5, HFI1, SPT20) is a subcomplex of all SAGA, SALSA, and SLIK, which highlights the importance of considering nested groups and not only nested pairs.
- DNA replication is a process involving the sequential assembly of subcomplexes into a maximal complex: the origin recognition complex (ORC1, ORC2, ORC3, ORC4, ORC5, ORC6) binds at replication origins, followed by CDC6, CDT1, and the mini chromosome maintenance complex (MCM2, MCM3, MCM4, MCM5, MCM6, MCM7). The resulting maximal complex is called “prereplication complex”, and separates the DNA strands at the origin.
- A final example of subcomplexes as substructures in MIPS is the case of microtubules: MIPS contains tubulin associated proteins or MAPs (ASE1, ATG4, ATG8, BIK1, BIM1, CBF5, CIN2, CIN4, MHP1, RBL2, SPC98, STU1, STU2, YTM1), which are proteins that stabilize, destabilize, and guide microtubules to specific locations, among other functions. MAPs are considered as a subcomplex of microtubules (32 proteins), and, finally, microtubules are considered a subcomplex of the cyto-skeleton (73 proteins).

It is important to notice that some of the found nested complexes are spatially nested, or modular structures (e.g., microtubules), while others are temporally nested, or dynamic structures (e.g., cell cycle-related complexes). It is also important to notice that experimental methodologies to detect complex isoforms and subcomplexes have been developed such as [25].

### 3.2. Quantifying Overlap and Nesting in iRefIndex Complexes

**Figure 2:**
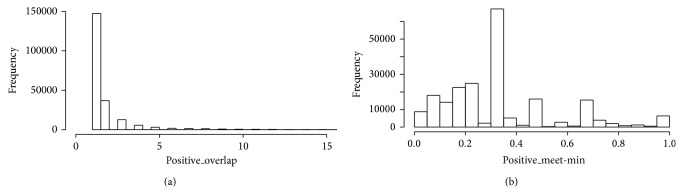
Distribution of overlap (intersection) and nesting (meet-min index) between every pair of iRefIndex protein complexes. (a) Overlap distribution. (b) Nesting distribution.

We have generated an “overlap matrix,” which is a matrix that includes the iRefIndex protein complexes as rows and columns and contains the information of the size of the intersection between every possible pair of complexes (number of common proteins). We found 199,665 pairs that display some degree of protein overlap, out of 16,247,850 possible pairs. We have also generated a “nesting matrix,” which contains the meet-min index, instead of the intersection. The meet-min is the quotient between the intersection and the size of the smaller of the two subsets under comparison, which means that a meet-min of zero corresponds to nonoverlap and a meet-min of one corresponds to perfect nesting. Here we found 8,145 nested pairs out of 16,247,850 possible pairs. The histograms in Figure 2 show the distribution of the intersection and meet-min indices. The overlap seems to occur with the intersection of a few proteins, while perfect nesting is not common (only 8,145 cases).

### 3.3. Representation and Visualization of Nested Complexes

**Figure 3:**
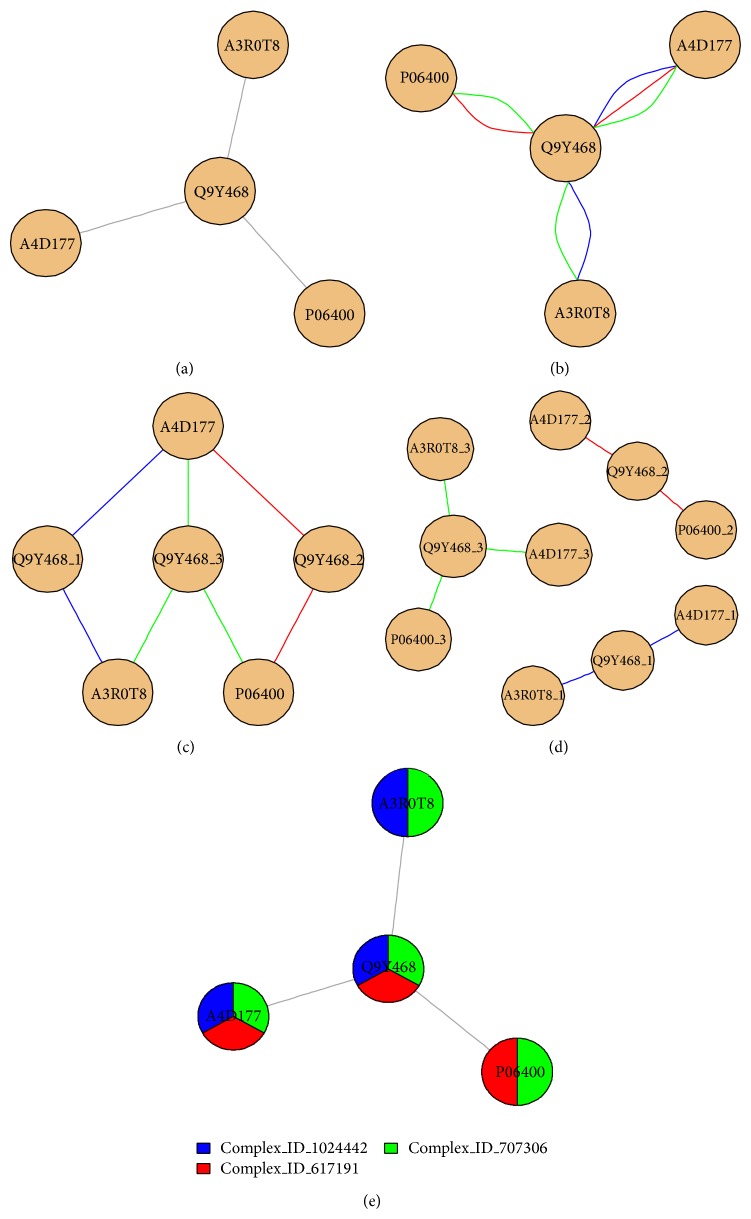
Representation and visualization methods of nested complexes. (a) Maximal complex, hiding nesting. (b) Multiedge representation. (c) Multihub representation. (d) Multinode representation. (e) Node-attribute representation. The multiedge view keeps one node per protein and represents every different complex with a different edge color. The multihub view splits the hub of the complex into three different nodes, one for each complex. The multinode just generates three separated complexes (distinct nodes and edges in the same graph). The node-attribute view uses single edges and, here, stores the information on complex membership as a pie for each node.

A single-edge representation of interaction networks (Figure 3(a)) hides the information regarding nesting and, therefore, we have considered four strategies to represent and visualize nested complexes.

1. Multiedge representation: in this case (Figure 3(b)), multiple edges mean membership to multiple complexes; here, we keep one node per protein and each edge color represents a different complex. This is the representation used in Figure 1.
2. Multihub representation: in this case (Figure 3(c)), the central hub was split into three nodes, one node per complex; as a result, we get single edges and a clear view of each complex, but the number of nodes has been artificially altered.
3. Multinode representation: in this case (Figure 3(d)), nodes are multiplied: each node (not only the hub) is split into one node per complex. As a result, we get the plot of all three disconnected complexes.
4. Node-attribute representation (Figure 3(e)): a way to keep the traditional single-edge plot is to not store complex information in the edges but in the nodes. In this case, each node becomes a pie plot.

Each of these methods has limitations. The multiedge representation can make the visualization very complicated due to the presence of more edges, but traditional network analysis metrics such as degree and centrality can still be applied to the nodes. The multihub and multinode views artificially expand the number of nodes. Finally, the node-attribute representation can be good for visualization purposes but hides the edge information from the abovementioned network metrics.

Choosing a representation is not only a visualization problem. This decision will have an impact on the measured network properties and, therefore, all conclusions will be relative to that chosen representation. From now on, we will assume a multiedge representation of the networks: metrics such as degree and betweenness centrality can still be computed for a multiedge graph, but we must bear in mind that some of the edges will link to different proteins while some edges will link to the same protein in a different complex.

### 3.4. Analysis of Proteins and Complexes in Nested Groups

#### 3.4.1. Naive Analysis of Nested Groups

**Figure 4:**
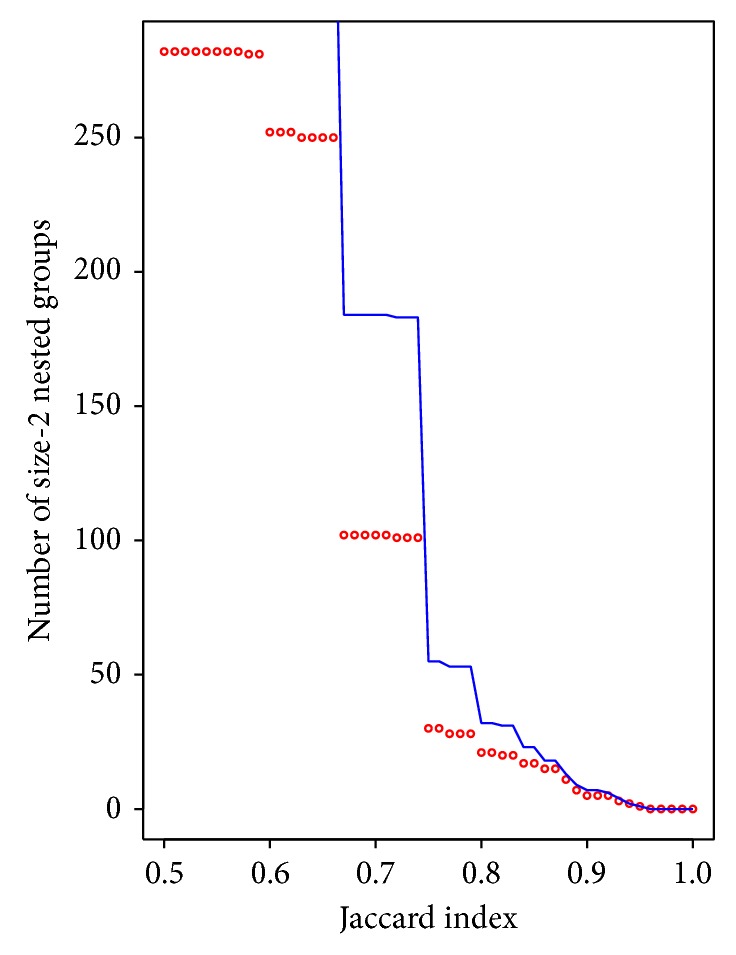
Number of size-2 nested groups (one maximal complex and one subcomplex) for a given Jaccard index. The red line represents the whole subset while the blue dots represent just the number of cases coming from more than one paper or database. High Jaccard seems to correlate with information coming from multiple sources, suggesting that high Jaccard in nested groups may come from data integration problems.

It could be hypothesized that a high Jaccard index (i.e., nested groups sharing most proteins and nonsharing one or a few) is related to artifacts or mistakes. In order to test this hypothesis, we studied all nested groups with a given Jaccard index and evaluated if the complex and subcomplex were found from two different papers or databases (suggesting an information integration problem).  Figure 4 shows that, when Jaccard values increase, the number of cases coming from multiple sources (red dots) is closer to the total number of cases with that Jaccard index (blue line). For example, for Jaccard = 0.75, 30 out of the 55 cases belong to more than one paper or database, and, for Jaccard >0.9, all cases come from more than one source. This is suggesting that high Jaccard in nested groups could be related to information integration problems.

A second hypothesis is that a high number of subcomplexes in a nested group is an indicator of real subcomplexes, as having several mistakes in a group is less likely than having one or two. In order to test this, we compared the number of subcomplexes in a gold standard subset (CORUM database) to all nested groups (based on iRefIndex), expecting to find a higher number of subcomplexes in the gold standard. However, we found only 170 out of 750 (22.7%) nested groups with at least three subcomplexes in CORUM, versus 398 out of 1427 (27.9%) in iRefIndex. Therefore, CORUM nested groups are not richer in subcomplexes than the consolidated data set. High number of subcomplexes is not a feature of the gold standard and, therefore, may not be related to real subcomplexes. This can be corroborated with a hypergeometric test (*P* value = 1). Therefore, multiple subcomplexes also seem to indicate a mistake or an artifact instead of real nesting. Based on this, the analysis in Figure 4 was made using only a reliable set of nested complexes (Jaccard > 0.85).

#### 3.4.2. Network Analysis (Degree, Centralities, and Clustering Coefficient) of Nested Groups

It has been reported that multiclustered proteins have higher degree and centralities than monoclustered [5]. Based on the assumption that nesting is an extreme case of overlap, we have tested those claims. We computed degree, betweenness centrality, page rank centrality, and clustering coefficient for all shared proteins (potentially present in more than one complex of the nested group) and nonshared proteins (present in the maximal complex only) of the human interaction network, and then we compared the values of each metric and each set (shared and nonshared) through a Wilcoxon test. The results show that all four metrics are significantly higher in shared proteins than in nonshared, as it can be observed in Table 1. Besides this, the proteins with the highest degree, betweenness, page rank, and clustering coefficient are always shared proteins and this situation does not change if we remove all multiple edges from the network.

#### 3.4.3. Essentiality and Relationship to Cancer

As we mentioned before, subcomplexes have been reported to be enriched in essential proteins, and products of cancer genes have been reported to be enriched among multiclustered proteins. Our analysis of essentiality in our consolidated data set (see Section 2) has confirmed that subcomplexes are significantly enriched in essential proteins. Moreover, nonshared proteins are also significantly enriched in essential proteins (*P* values approximately zero). On the other hand, a negative result was found in terms of cancer enrichment. We ran a hypergeometric test (see Section 2) and, as a result, neither shared nor nonshared proteins display statistically significant enrichment in cancer proteins.

#### 3.4.4. Motif Analysis

**Figure 5:**
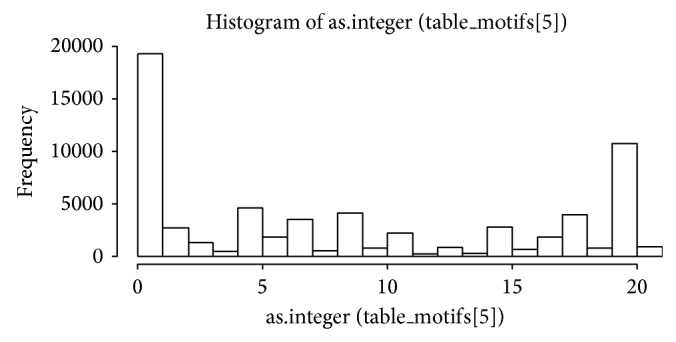
Histogram of number of found motifs per nested group.

We also studied all possible undirected motifs involving 3, 4, or 5 proteins. The motifs were generated for each nested group as explained in Section 2, and the number of motifs and statistically significant motifs were recorded. In total, 64,027 motifs were found in our nested groups, 46,088 of which (72%) were statistically significant. These were mainly 5-motifs, followed by 4-motifs and then 3-motifs. A histogram of the motif distribution shows a slightly bimodal distribution, with peaks around 0 and 20 motifs per nested group, as shown in Figure 5.

**Figure 6:**
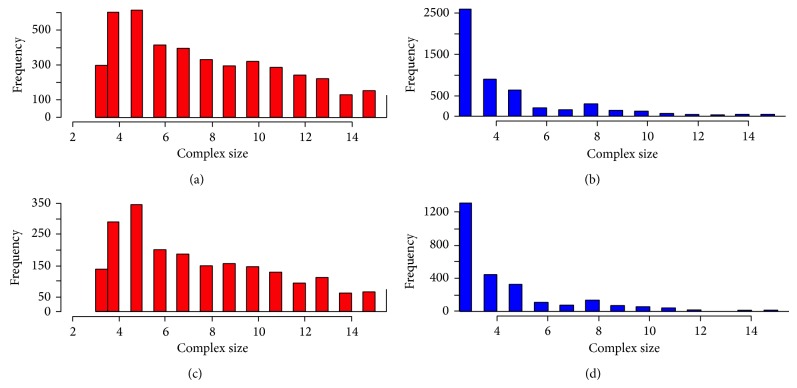
Histogram of complex size in maximal complexes and subcomplexes. (a) Size distribution of the maximal complexes in the full set of nested complexes. (b) Size distribution of subcomplexes in the full set of nested complexes. (c) Size distribution of the maximal complexes in the reliable set of nested complexes. (d) Size distribution of subcomplexes in the reliable set of nested complexes.

A study of the most common motifs shows that this is the imperfect 5-clique, that is, a clique of 5 proteins with one, two, or more missing edges. This is evidence in favor of relating the concept of nested complexes to the concepts of densely connected groups such as cliques or cographs, as has been traditionally done in protein complex detection. In order to unveil the meaning of this enrichment, we generated histograms of the complex size distribution of maximal complexes and subcomplexes, as shown in Figure 6.

As a result, we found that maximal complexes are mainly 5-protein complexes (and up to 220 proteins), while subcomplexes mainly contain 3-4 proteins. That means every time that a subcomplex has a different hub than the maximal complex in a spoke model, it is probable to find an incomplete 5-clique motif. Therefore, incomplete 5-cliques are a signature to find nested complexes represented as spoke models using a different hub.

#### 3.4.5. Modularity Analysis in Nested Groups

We evaluated whether some traditional community detection algorithms would still be able to find communities inside nested groups, and, if so, if those communities had a good modularity value. In order to do that, we applied three algorithms to every nested group: edge betweenness communities, walktrap communities, and label propagation communities [26–28].

**Figure 7:**
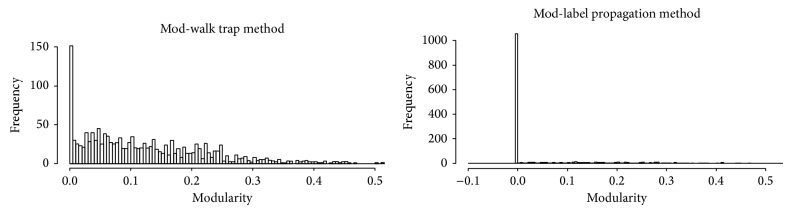
Modularity distribution following the community detection for all nested groups. Modularity values are generally poor while, in most cases, a further subdivision of the nested group is not possible.

The first result was that, in general, trying to find communities in nested groups is not possible in many cases and, when possible, do not lead to good modularity values, as shown in Figure 7. Most cases display a modularity of zero and, besides that, there are a small number of cases with small modularity values. The second result is that we can find very few cases where the generated communities behave as good predictors of the subcomplexes. In order to do that, for each nested group, we recorded the predicted communities and the known subcomplexes and counted the true positives, false positives, and false negatives. With these measures, we computed the precision and FDR for each case. The results were mainly low precision and high FDR, even though many predictions happened to be correct.

#### 3.4.6. GO Terms Analysis in Nested Groups

It has also been reported that multiclustered proteins are, on average, annotated to more terms than monoclustered and are enriched in biological process GO terms [6] referring to regulatory functions and protein complex assembly [5]. And that subcomplexes are more homogeneous in terms of the GO terms of their subunits [8].

Regarding the first hypothesis, Table 2 shows the number of GO terms found per shared and nonshared proteins, per each of the three GO ontologies. There are 2,239 shared proteins versus 17,243 nonshared proteins. Therefore, the value of the nonshared proteins was calculated using Monte Carlo simulations (average of 1,000 simulations), where each of them contains 2,239 nonshared uniformly randomly chosen proteins. The table shows that there are slightly more terms in shared proteins for the BP and CC ontologies, while this is not true for the MF ontology.

## 4. Conclusion

This paper provides a novel characterization of nested complexes. We introduced the concept of “nested group”: a nested group is a group of complexes formed by one maximal complex and one or more subcomplexes. A subcomplex was defined as a complex whose nodes are a subset of the nodes of the maximal complex, while its edges do not need to be a subset of the maximal complex's edges. In theory, such nested groups are related to the “hierarchical organization” concept, but, in practice, complex representation issues made them appear as either hierarchies or as overlapping complexes in a protein interaction network.

Using the consolidated protein interaction database iRefIndex, we generated 1,427 nested groups in the human interactome. The complex members of 189 out of the 1,427 generated nested groups were found to come entirely from the same paper and the same database, which can be interpreted as researchers being aware of the existence of both the complex and the subcomplex, instead of being an experimental or bioinformatics artifact. Besides that, it was easy to find examples in both the CORUM and MIPS databases of nested groups in human and yeast with annotation indicating biological relevance, such as substructures of a macrostructure (nuclear pore complex), functional processes such as DNA repair, diseases such as cancer, and dynamic complexes such as those related to the cell cycle. Therefore, complex nesting can be used as a unifying framework to deal with both structure-substructure data and dynamic interaction data.

We could observe that protein overlap seems to happen most of the time in the interactome with the intersection of a few proteins, while perfect nesting is not a common phenomenon. We also observed that predicting subcomplexes using popular community detection algorithms (to find densely connected subnetworks inside a densely connected network) did not work as expected. Subcomplexes dont have an especially good modularity and are not equivalent to the nested communities found by some community detection algorithms.

However, several characteristics that allow us to identify a nested complex were found: in the first place, when Jaccard index values increase, the number of cases coming from multiple sources (papers and databases) is closer to the total number of cases with that Jaccard index. This is suggesting that high Jaccard index in nested groups could be related to information integration problems and allows us to define a high-confidence subset of nested groups. Second, network metrics such as degree, betweenness centrality, page rank centrality, and clustering coefficient proved to be useful to distinguish between shared (nested) and nonshared (nonnested) proteins. Third, the most common motif in nested groups happens to be the imperfect 5-clique, which comes from a size-5 complex with a size-3 or size-4 subcomplex, using a different protein as hub of the spoke model. Fourth, both shared and nonshared proteins of a nested group are essential proteins. That means that not only the subcomplexes, which may be evolutionary conserved reusable modules, are essential, but also the rest of the nested group. Finally, nested or shared proteins are, on average, annotated to more BP and CC GO terms than nonnested or nonshared, and their GO terms are more homogeneous.

The previous information may lead to promissory strategies in order to design an algorithm that predicts nested groups or subcomplexes. Such an algorithm could make use of the identified strategies, for example, (a) detecting multiple edges and hypothesizing a nested group, (b) identifying misrepresented overlapping complexes, (c) fixing missrepresentation by changing the complex representation model, (d) filtering out all nested groups with a high Jaccard index, (e) applying a community detection algorithm to find densely connected regions, and (f) recording the results in a multiedge representation. Besides contributing to the study of hierarchically organized data in interaction databases, we expect such algorithm to be important to identify macrocomplex structures or dynamic complexes.

## Figures and Tables

**Table 1:** Network properties of shared and nonshared proteins.

	Shared proteins	Nonshared proteins	*P* values
Avg. degree	73.4	27.2	7*e* − 183
Avg. betweenness centrality	145647.5	41680.9	4*e* − 95
Avg. page rank centrality	0.0002	7*e* − 5	1*e* − 168
Clustering coefficient	0.20	0.17	3*e* − 30

**Table 2:** Number of GO terms for each of the three gene ontologies, for shared and nonshared proteins, in nested groups.

	Shared proteins	Nonshared proteins-mean	Nonshared proteins-sd
Biological process (BP)	3572	3313.8	91.8
Molecular function (MF)	916	1130.9	30.1
Cellular component (CC)	700	526.2	16.0
